# Nanometers-Thick Ferromagnetic Surface Produced by Laser Cutting of Diamond

**DOI:** 10.3390/ma15031014

**Published:** 2022-01-28

**Authors:** Annette Setzer, Pablo D. Esquinazi, Sergei Buga, Milena T. Georgieva, Tilo Reinert, Tom Venus, Irina Estrela-Lopis, Andrei Ivashenko, Maria Bondarenko, Winfried Böhlmann, Jan Meijer

**Affiliations:** 1 Division of Superconductivity and Magnetism, Felix-Bloch-Institute for Solid State Physics, University of Leipzig, 04103 Leipzig, Germany; setzer@rz.uni-leipzig.de (A.S.); mgeorgieva@phys.uni-sofia.bg (M.T.G.); bohlmann@physik.uni-leipzig.de (W.B.); 2 Technological Institute for Superhard and Novel Carbon Materials, 7a Centralnaya Street, 108840 Moscow, Russia; sergei_buga@mail.ru (S.B.); iva_andrey@inbox.ru (A.I.); maria7273@mail.ru (M.B.); 3 Division of Applied Quantum Systems, Felix-Bloch-Institute for Solid State Physics, University of Leipzig, 04103 Leipzig, Germany; tilo.reinert@uni-leipzig.de (T.R.); jan.meijer@uni-leipzig.de (J.M.); 4 Institute of Medical Physics and Biophysics, University of Leipzig, 04107 Leipzig, Germany; tom.venus@medizin.uni-leipzig.de (T.V.); irina.estrela-lopis@medizin.uni-leipzig.de (I.E.-L.)

**Keywords:** diamond, magnetic order, laser treatment

## Abstract

In this work, we demonstrate that cutting diamond crystals with a laser (532 nm wavelength, 0.5 mJ energy, 200 ns pulse duration at 15 kHz) produced a ≲20 nm thick surface layer with magnetic order at room temperature. We measured the magnetic moment of five natural and six CVD diamond crystals of different sizes, nitrogen contents and surface orientations with a SQUID magnetometer. A robust ferromagnetic response at 300 K was observed only for crystals that were cut with the laser along the (100) surface orientation. The magnetic signals were much weaker for the (110) and negligible for the (111) orientations. We attribute the magnetic order to the disordered graphite layer produced by the laser at the diamond surface. The ferromagnetic signal vanished after chemical etching or after moderate temperature annealing. The obtained results indicate that laser treatment of diamond may pave the way to create ferromagnetic spots at its surface.

## 1. Introduction

Since the first studies on the magnetic order found in pure graphite-based samples were reported, see [1] and Refs. therein, the possibility of having magnetic order in other carbon-based compounds at room temperature and without doping with magnetic ions attracted the interest of the community. In case of pure diamond, Talapatra et al. [2] reported the existence of ferromagnetic hysteresis at room temperature in the magnetization of nanograins of diamond after nitrogen and carbon irradiation. This interesting result was ascribed to structural modification or defects produced by the irradiation, a clear case of the phenomenon called defect-induced magnetism (DIM). In contrast to the ${}^{12}$C implantation, a higher value of the magnetization at saturation was obtained after ${}^{15}$N implantation, which was interpreted as due to the extra contribution of N-related centers in the diamond crystalline structure [2]. Superconducting (with a transition temperature of $T_{c}∼3\;$K) and ferromagnetic (Curie temperature of $T_{C}>400\;$K) states were found in hydrogenated boron-doped nanodiamond films by Zhang et al. [3]. Narayan and Bhaumik reported ferromagnetic states after quenching carbon from an undercooled state using nanosecond laser pulses [4]. The observed magnetic state at room temperature, which depended on the energy and number of laser pulses, was attributed to a mixture of sp${}^{2}$–sp${}^{3}$ bonds in the nanostructure of the diamond samples. Theoretical work studied the possibility of ferromagnetism in diamond taking into account disorder and certain doping [5]. As in other carbon-based structures [6,7,8,9,10,11,12,13], H-atoms or H${}^{+}$ in the diamond lattice might also trigger a finite magnetic moment, although the influence of their position in the diamond lattice on the magnetism has to be still clarified [5].

In this work, in contrast to the above mentioned studies about triggering magnetic order in the diamond structure, we are mainly interested to study the possible development of magnetic order through a graphitization of the diamond surface via laser pulses. Several experimental and theoretical studies on the origin of ferromagnetism in graphite without magnetic impurities have been published over the last 20 years; for reviews, see Ref. [1]. With a density of lattice defects or hydrogen between 5% and 10%, graphite can be magnetically ordered with a strong spin-polarized valence band, which affects the polarization of the barely occupied conduction band (graphite is a narrow-band-gap semiconductor [14,15]).

It has been known for almost 20 years, see [16] and Refs. therein, that the surface of pure diamond can be graphitized via laser pulses. The heating of the diamond under the influence of laser radiation leads to graphitization, ablation and burn of carbon material [16,17,18]. The characteristics of the graphite structure at the surface of the diamond sample (e.g., defects density, crystal orientation, etc.) partially depend on the crystal orientation and length of the laser pulse [16,18]. In a recently published work, the effect of the cutting fluence (of a 532 nm wavelength laser with a pulse duration of 40 ns and a spot diameter of 40μm) on a CVD diamond surface was investigated with Raman and transmission electron microscopy (TEM) [19]. The authors found that the subsurface of the diamond samples showed a mixture of graphite and amorphous carbon and that the thickness of the graphite layer decreased with laser fluence. Systematic studies on this topic have been published earlier [20]. However, no magnetic characterization of the produced graphite/amorphous carbon layers was reported. In this study, we used laser pulses of 532 nm wavelength, 300 J/cm${}^{2}$ energy density in a 15μm focus spot and 200 ns pulse duration at 15 kHz to produce a graphitic-like layer at the surface of several diamond samples and studied their magnetic properties.

## 2. Samples and Methods

### 2.1. Laser Cutting and After-Cutting Processes of Diamond Crystals

A single crystal of diamond was glued to the base surface of a mandrel, so that a large face was orientated perpendicular to the axis of the mandrel. Next, the mandrel (with the diamond crystal) was fixed in a device for the precise positioning in the laser-cut system. The system was equipped with a video camera that allowed us to adjust the face of the crystal to be cut along the axis of the laser beam to achieve the shortest laser cut length. The marking of the cut line on the selected face of the diamond single crystal was carried out on the computer monitor with the help of optical devices.

**Figure 1:**
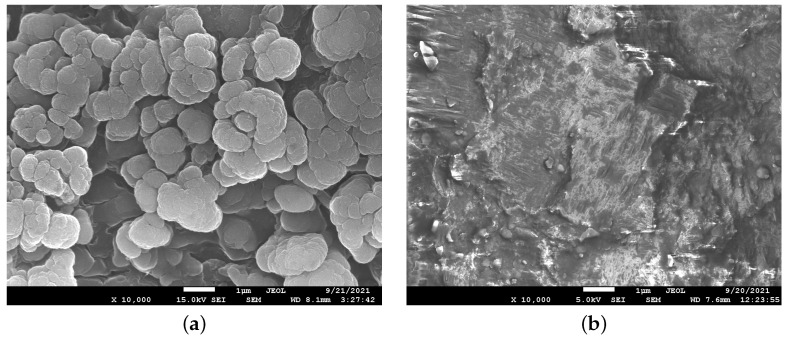
Scanning electron microscope images of the surface of a CVD diamond sample after laser cut (**a**) and after laser polishing (**b**).

Before cutting, the laser beam was focused on the surface of the diamond at the level of the upper point of the cut. Then, the laser beam was moved along the cut line, where the material was burned on the surface of the diamond with a width nearly the diameter of the laser beam focus. Thus, the working pass was performed at a certain depth in the crystal. After leaving the diamond sample, the laser beam was moved by a step (specified in software) in the transverse direction and then moved in the opposite direction performing the next working pass. By selecting the wavelength, power and the duration of the laser radiation pulses, we could control the volume of material removal. A SEM image of the obtained surface can be seen in Figure 1a. Figure 1b shows the obtained surface after polishing the cut face by focusing the laser beam with the specified parameters directly on its surface. In this way, the laser beam burned off part of the surface material produced by the cutting.

In order to remove the graphitic-like nanometer-thick ferromagnetic surface region formed after laser treatment, we used two methods. (1) The first method consisted of chemical etching of the laser cut samples with a mixture of 30 mL of concentrated sulfuric acid (H${}_{2}$SO${}_{4}$), 10 mL of fuming salpetric acid (HNO${}_{3}$) and 10 mL of 70 vol% perchloric acid (HClO${}_{4}$); this mixture was heated at 120 ${}^{\circ }$C for 4 h. under reflux. After cooling to room temperature, the acids were decanted and the diamond was intensively washed with distilled water and dried with nitrogen gas. In comparison to the literature [21,22], we applied a modified etching procedure at higher temperatures with a mixture of strong oxidizing acids to remove the graphite residues derived from the laser treatment. With this procedure, we estimated that the disordered graphite thickness that the etching process removed should have been at least ∼20nm. Certainly, not all this thickness might be magnetically ordered. From a comparison between the magnetization at the saturation values of ferromagnetic graphite [1,11] and our laser-treated samples, we estimated that the ferromagnetic thickness should have been ≲20nm; see Section 3.3. (2) The other method we used was annealing the diamond sample in air at temperatures T≤650 ${}^{\circ }$C for a couple of hours.

### 2.2. Samples Characteristics

#### 2.2.1. Natural Diamond Samples

Table 1 shows several characteristics of the natural diamond samples, such as the orientation of the laser-cut surface, as well as the total nitrogen concentration N and the following nitrogen-related defect concentrations: -A, a neutral nearest-neighbor pair of nitrogen atoms substituting the carbon atoms; -B, a carbon vacancy surrounded by four nitrogen atoms substituting the corresponding carbon atoms. -C, electrically neutral single substitutional nitrogen atoms in the diamond lattice, sometimes called also P1-center—see, e.g., [23,24].

#### 2.2.2. CVD Diamond Samples

As we demonstrate below in this paper, the orientation of the laser-cut surface plays a main role in producing the robust ferromagnetic nanometer-thick surface region at room temperature. To support the results obtained from the natural diamond crystals, we cut 6 CVD diamond samples at 3 orientations (100), (110) and (111); see Table 2. These samples had a total concentration of magnetic impurities below 2 ppm and a much lower N concentration (≲10ppm) than the natural diamond samples. We measured the magnetic response of the CVD samples after the first cut (state “a”) and after polishing the cut surface with the laser beam (state “b”). There was basically no difference in the magnetic behavior between the states “a” and “b”, which we expected after removing part of the cut surface by polishing the ferromagnetic signal as the saturation became smaller.

### 2.3. Methods: PIXE, Raman and SQUID Characterization

The quantitative characterization of the main magnetic impurities (Fe, Co and Ni) was performed using particle-induced X-ray emission (PIXE) with protons. The parameters were the following: proton energy, 2.0 MeV; current, 2.5 nA; slit settings, object/aperture of 300 μm/300 μm and a beam focus of 3 μm. Protons of 2 MeV kinetic energy have a penetration range in diamond of about 25 μm. The X-ray production cross-section decreases as the protons slow down inside the sample. Additionally, the contribution of X-rays to the detectable analytical signal decreases with depth. However, this effect for X-rays from Fe, Co and Ni is less important due to the rather short range of 2 MeV protons in a carbon matrix.

Confocal Raman measurements were performed with a WiTec Alpha 300 System. A laser wavelength of 532 nm (UHT S 300) was selected. A 50× objective (Zeiss, Germany) with a numerical aperture of 0.8, a laser power at the sample surface of ca. 35 mW and a 1800 grating on the CCD detector with spectral resolution of ca. 0.8 cm${}^{-1}$ were used.

The measurements of the magnetic moment of the diamond samples were performed with a superconducting quantum interferometer device (SQUID) from Quantum Design. Magnetic field loops and temperature hysteresis were obtained after demagnetizing the samples at 380 K. The time between two consecutive measurements at different fields or temperatures was 5 min or longer with similar results. No time dependence was detected within the experimental resolution.

## 3. Results

### 3.1. Magnetic Impurities Measurements

From the characterization of the impurities with PIXE, we conclude that the maximum magnetic impurity concentration was 2.6 ppm of Fe in sample 164. Sample 354, which showed the largest magnetization at saturation, had a total concentration of magnetic impurities below 0.5 ppm; see Table 3.

As an example, let us estimate how large would be the contribution of 0.17 ppm Fe in sample 354 to the magnetization at saturation. We assume that this small concentration of Fe or, most likely, magnetite, Fe${}_{3}$O${}_{4}$, behaves as a bulk ferromagnet with a saturation magnetization of about 100 emu/g. The measured concentration of Fe in sample 354 would imply a ferromagnetic total mass of 76 ng, which, in the unrealistic largest case, could contribute with a magnetic moment of 4.8μemu. This value is 4.2 times smaller than the magnetic moment at saturation measured at 300 K (see Section 3.3.1 below).

### 3.2. Raman

**Figure 2:**
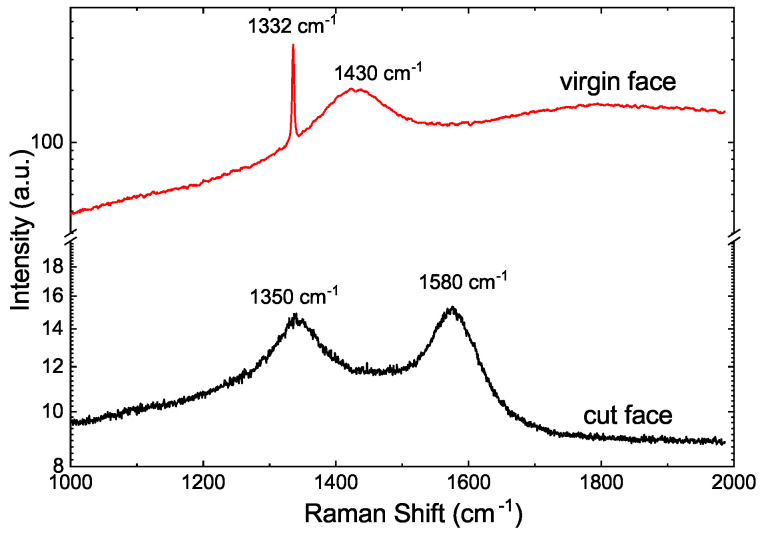
Raman spectra obtained at room temperature for the virgin and laser-cut faces of the CVD sample #1b.

Raman measurements were performed on the “virgin” surface, i.e., a region of the same sample without any laser treatment, and on the laser-cut surfaces of all samples. As an example, we show, in Figure 2, the results of the CVD sample #1b. Whereas the virgin surfaces of the samples showed a sharp absorption peak at 1332 cm${}^{-1}$, corresponding to pure diamond (first order Raman), the laser-cut surfaces showed disordered graphite-like peaks due to the G-band (1580 cm${}^{-1}$) and D-band (1350 cm${}^{-1}$); see Figure 2. In the case of the virgin surfaces, the peak at 1430 cm${}^{-1}$ observed in the CVD samples [25,26,27], was also clearly observed; see Figure 2.

The differences in the Raman patterns between the CVD “a” and “b” samples’ cut surfaces were the following. The Raman spectrum of the “b” samples corresponded to disordered graphite. However, the “a” cut surfaces also indicated a weak contribution of the diamond main Raman peak. We interpret this as the laser polishing transforming the rest of the diamond-like regions to disordered graphite regions. The experimentally observed broadening of the Raman peaks did not allow us to formulate a clear description on the presence of certain defects that could be correlated with the magnetic response.

### 3.3. Magnetization Measurements

#### 3.3.1. Natural Diamond Samples

**Figure 3:**
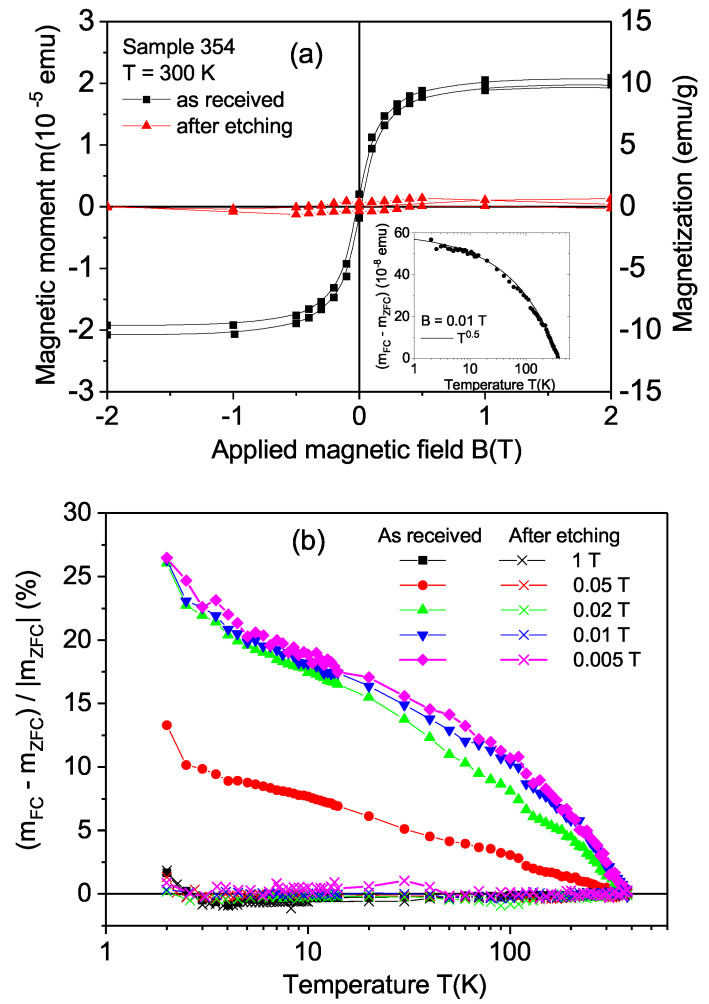
(**a**) Field hysteresis loops at 300 K of sample 354 in the as-received state and after chemical etching. The same linear diamagnetic background was subtracted from the measured curves. The inset shows the temperature dependence of the difference in the magnetic moment measured in the field-cooled (FC) and zero-field-cooled (ZFC) states measured at a fixed field of 0.01 T. The continuous line follows the equation $m_{FC}\left(T\right)-m_{ZFC}\left(T\right)=10^{-8}\left(60-3T^{0.5}\right)$ (emu). (**b**) Temperature dependence of the relative difference $100\left[m_{FC}\left(T\right)-m_{ZFC}\left(T\right)\right]/\left|m_{ZFC}\left(T\right)\right|$ before and after etching.

With the magnetic impurity concentration of our samples, the natural diamond crystals in the virgin state (before any laser treatment) did not show any sign of magnetic order at 300 K within the resolution of our SQUID magnetometer (∼$5\times 10^{-8}$ emu at an applied field of 1 T). Figure 3a shows the field hysteresis loops of sample 354 at 300 K before (as-received) and after chemical etching, within a ±2 T field range. The same diamagnetic linear contribution was subtracted from both data sets. Before chemical etching, the sample showed a clear ferromagnetic response. In the inset of Figure 3a, we plot the difference between the field-cooled (FC) and zero-field-cooled (ZFC) states at a 0.01 T applied field. This difference followed a temperature dependence similar to that found in irradiated graphite [1,28]. This similarity and the Raman results—see Section 3.2—indicate that the disordered graphite layer produced by the laser treatment should be at the origin of the observed ferromagnetic response. As a proof of this assumption, the same sample was treated chemically to remove the disordered graphitic layer. The reduction in the ferromagnetic response observed in the field hysteresis loop of Figure 3a after chemical etching clearly indicates that the ferromagnetic behavior was related to the graphitic-like layer produced by the laser treatment.

To further demonstrate the large difference in the ferromagnetic response between the cut sample before and after etching, the difference between the FC and ZFC states relative to the value in the ZFC state given by $100\left[m_{FC}\left(T\right)-m_{ZFC}\left(T\right)\right]/\left|m_{ZFC}\left(T\right)\right|$ at different applied magnetic fields is shown in Figure 3b. In this figure, we recognize that, whereas this relative difference reached ∼25% (of $|m_{ZFC}\left(T\right)|$) at low temperatures and at fields ≤0.02T in the as-received sample, it remained below 1% in the whole temperature range and applied fields after etching the sample. These results further indicate that those signals were related to the surface near the graphitic-like region.

With the estimated ferromagnetic thickness of ∼20nm and the measured area of the laser-cut surface, we obtained a magnetization (right y-axis in Figure 3a) at a saturation of 10 emu/g. A comparison with the values of the magnetization at the saturation obtained for ferromagnetic graphite [1,11], we note that this ferromagnetic thickness should be of the order or even smaller.

**Figure 4:**
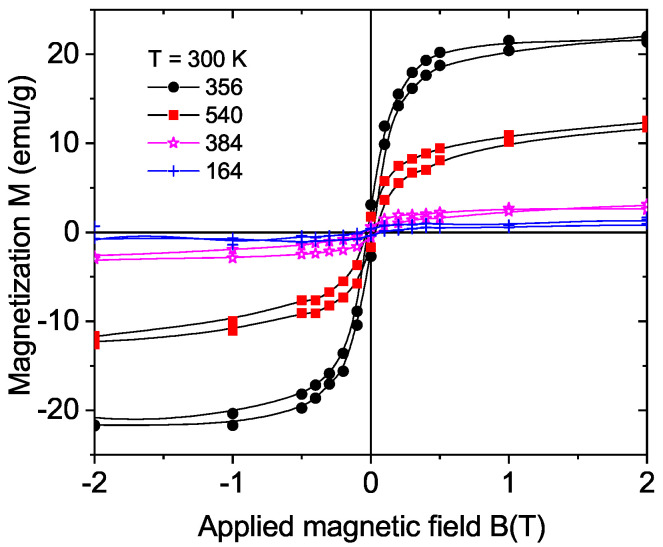
Field hysteresis loops at 300 K of four natural diamond samples ((100) surface, samples 356 and 540; (111) surface, samples 164 and 384). The magnetization values were calculated assuming a ferromagnetic mass given by a 20 nm thick region at the cut surfaces of the samples.

Figure 4 shows the field hysteresis loops of four natural diamond samples with cut areas with (100) orientation (samples 356 and 540, similar to sample 354; see Table 1) and with (111) orientation (samples 164 and 384) at 300 K. Taking into account the cut area and assuming the same ferromagnetic thickness, we recognize that the ferromagnetic signals were clearly smaller for the (111)-cut surface samples. This difference was not related to large differences in the assumed ferromagnetic mass because the cut surfaces were similar or their difference shifted the estimate value of magnetization in the opposite direction; see Table 1. This result indicates that the diamond crystalline structure and the laser cut direction relative to its structure played an important role in triggering the ferromagnetic order in the graphitic-like surface layer. The results obtained from the CVD samples support this conclusion; see Section 3.3.2.

**Figure 5:**
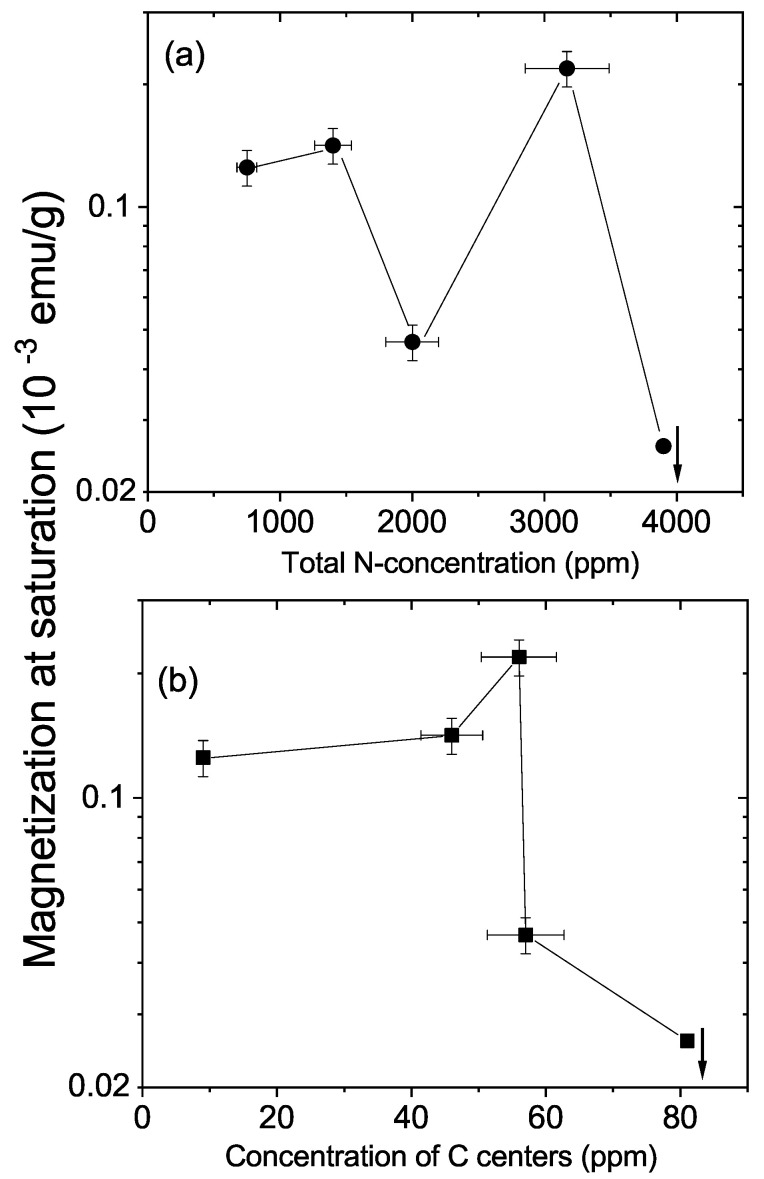
Magnetization at saturation at 300 K of the natural crystals vs. the total concentration of nitrogen (**a**) and of C centers (**b**). Because nitrogen was in all the sample volume, the magnetization was calculated taking into account the total mass of each sample. The down-arrow at the last point to the right means that the value of magnetization for that sample is below the limit of the y-axis.

Nitrogen doping with the concentrations measured in our samples, or lower—see Table 1—did not trigger ferromagnetic order at room temperature. The ferromagnetic signal was not related to the total N concentration or to the concentration of the three defect centers one finds in bulk N-doped diamond (A, B and C); see Figure 5a,b. In Figure 5a,b and due to the fact that these centers were distributed all over the samples, the shown magnetization values were obtained taking into account the whole sample mass. On the other hand, we note that N-related C centers were at the origin of the clear hysteretic behavior in field and temperature observed below 50 K [29,30].

#### 3.3.2. CVD Diamond Samples

**Figure 6:**
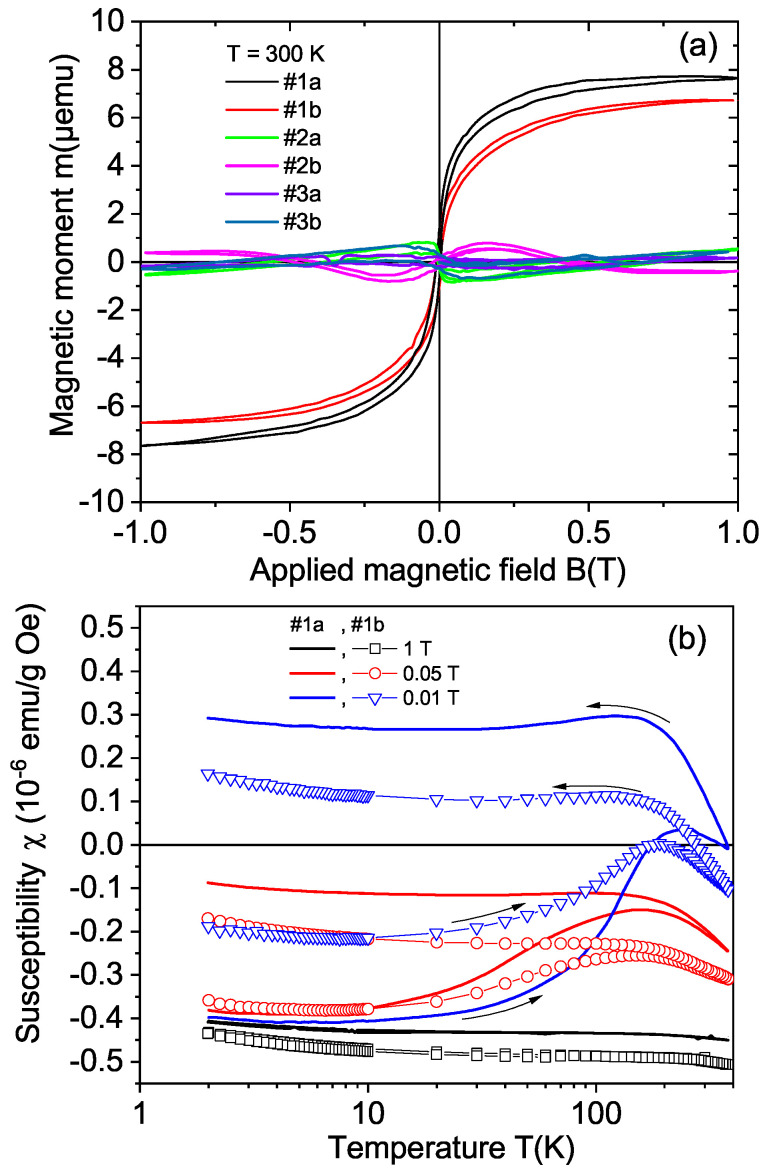
(**a**) Field hysteresis loops of the magnetic moment of all CVD samples at 300 K. The diamagnetic background was subtracted from the measured data. We note that the field loops obtained for some of the CVD samples were influenced by the hysteresis of the superconducting solenoid. This is a systematic error of the SQUID system that can influence the hysteresis loops. (**b**) The susceptibility as a function of temperature at constant fields in the zero-field-cooled (measurement by warming the sample after applying the corresponding fields at the lowest temperature) and field-cooled (measurement by cooling the sample at the same field) states of the two samples (#1a, continuous lines; #1b, symbols). No background was subtracted from the data. The mass used to calculate the susceptibility was the total sample mass, which was similar in both samples.

**Figure 7:**
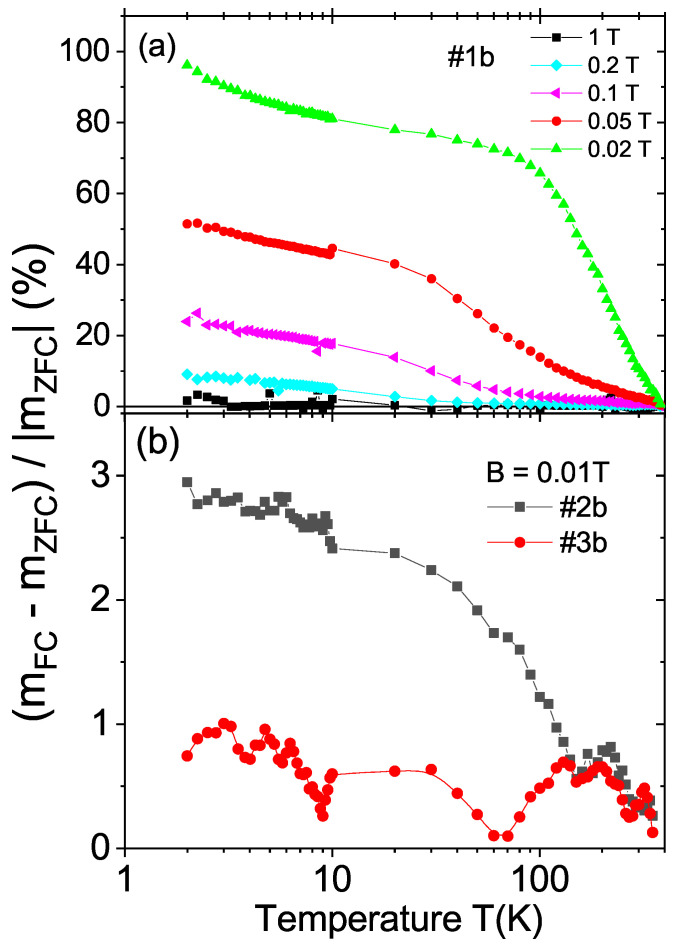
Temperature dependence of the relative difference $100\left[m_{FC}\left(T\right)-m_{ZFC}\left(T\right)\right]/\left|m_{ZFC}\left(T\right)\right|$ for the CVD samples: (**a**) #1b at five different applied fields and (**b**) #2b and #3b at a field of 0.01 T.

Figure 6a shows the field hysteresis loops measured at 300 K of all CVD samples—see Table 2—after subtracting the linear diamagnetic background. The results indicate a ferromagnetic behavior with a coercive fields of the order of 80 Oe for samples #1a and #1b. The magnetic moment at saturation was much smaller for the samples with other crystal orientations. Taking into account the volume of the cut surface or the total mass, the obtained ferromagnetic magnetization of samples #1a and #1b was always larger than that of the other CVD samples, supporting the orientational dependence of the ferromagnetic signals of the laser-cut surface observed in the natural diamond crystals. As in the natural diamond crystals, the samples cut with orientation other than the (100) showed a much smaller or negligible ferromagnetic signal; see Figure 7. The relative difference between the FC and ZFC curves was nearly two orders of magnitude larger for samples with (100)-cut surfaces.

We observe that the saturation magnetic moment of sample #1b obtained after laser polishing the cut surface was about 10% smaller than that of sample #1a. The ferromagnetic behavior was clearly observed in the difference between the ZFC and FC states, as shown by the temperature dependence of the susceptibility, see Figure 6b. As expected for a ferromagnetic behavior, the difference between the ZFC and FC states as a function of temperature vanished at high magnetic fields, in agreement with the vanishing of the field hysteresis width at high-enough fields; see Figure 6a.

**Figure 8:**
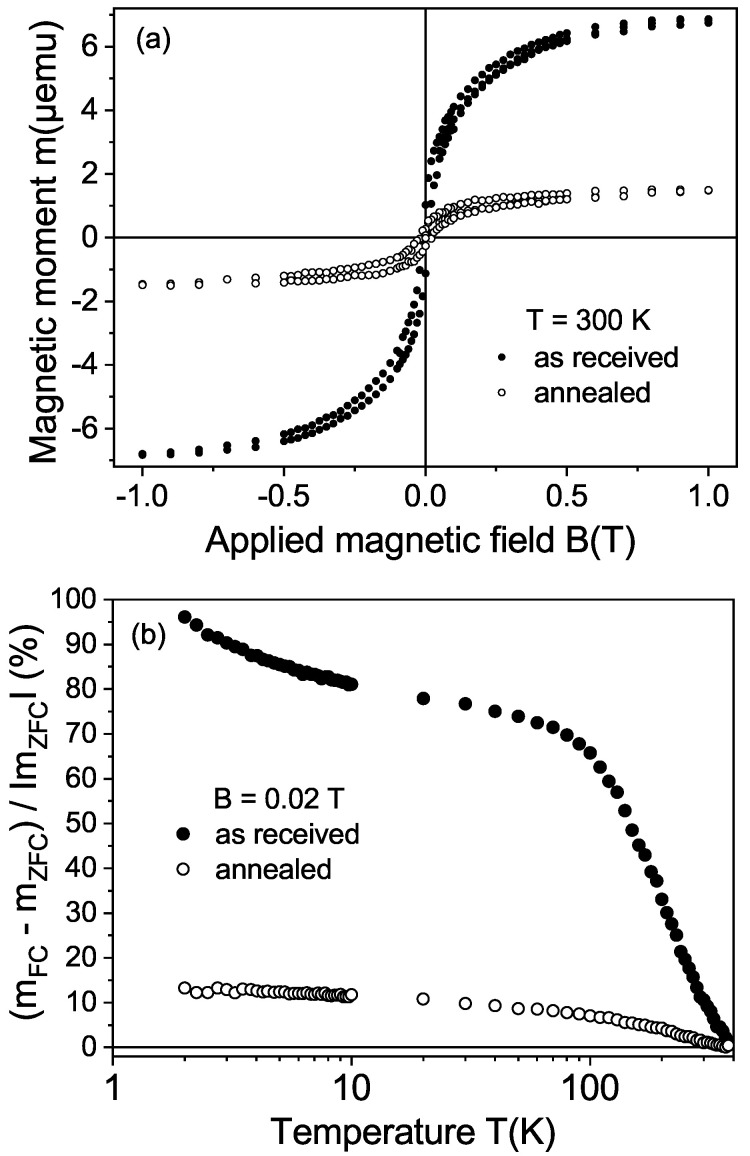
Magnetic properties of sample #1b: (**a**) Field hysteresis loops at 300 K in the as-received state and after the high-temperature annealing. (**b**) Temperature dependence of the relative difference $100\left[m_{FC}\left(T\right)-m_{ZFC}\left(T\right)\right]/\left|m_{ZFC}\left(T\right)\right|$ at a field of 0.02 T, before and after annealing.

It is known that high temperature annealing in air removes any graphitic-like surface regions in diamond. Therefore, instead of using chemical etching to remove the graphitic surface of the CVD samples, as performed in the natural diamond samples (see Figure 3), we annealed one of the CVD samples (#1b) in air. The annealing procedure in air was 1 h at 550 C, 1 h at 600 C and 0.5 h at 650 C. Similarly to the result after chemical etching of a natural diamond sample, the ferromagnetic signal strongly decreased after annealing; see Figure 8. All these results clearly indicate that the ferromagnetic signal came from the disordered graphite surface region obtained after the laser cut and it was not related to magnetic impurities.

Before concluding, we would like to remark that the Curie temperature of the ferromagnetic order observed in the laser-treated surfaces of diamond with the (100) direction was clearly larger than 380 K. The temperature of 380 K is the turning point temperature at which the FC measurement starts. For this reason, the difference $m_{FC}\left(T\right)-m_{ZFC}\left(T\right)$ is always zero at the turning-point temperature. A rough extrapolation of the observed temperature dependence of the magnetic moment to temperatures above 400 K—see, for example, Figure 4b and Figure 6b—indicates a Curie temperature between 500 K and 750 K, similar to defect-induced ferromagnetic graphite; see [1] and Refs. therein.

## 4. Conclusions

Independently of the origin of the diamond sample, natural or CVD, we found that, under the selected conditions, the laser pulses produced a robust magnetically ordered graphite film at 300 K in samples cut along the diamond (100)-surface orientation. Assuming a maximum thickness of 20 nm for the magnetic layer, the magnetization value at saturation varied from ∼10emu/g to 20 emu/g at 300 K, similar to the magnetization values obtained for defect-induced ferromagnetic graphite [1,11]. This magnetic order is clearly weaker or absent in the cases of the other two surface orientations. Further focused experimental characterization but also computer simulations, as in Ref. [17], are necessary to find the lattice defects (e.g., C-vacancies, sp${}^{2}$-sp${}^{3}$, or C-H complexes) responsible for the observed ferromagnetism. Laser treatment can, in principle, be used to create localized magnetic spots of small areas on a diamond surface. This phenomenon can be of interest not only for memory devices but also for other rather subtle applications, such as using a localized magnetic spot near a nitrogen–carbon vacancy (NV-center) to influence its magneto-optical response, especially to increase its field-sensitivity at certain applied field ranges.

## Figures and Tables

**Table 1:** Sample name, surface orientation, mass, cut area, total nitrogen concentration (N) and concentrations of the A, B and C centers of the natural diamond samples.

Name	Orientation	Mass	Cut Area	N	N Centers
		(mg)	(mm^2^)	(ppm)	(ppm) (A, B, C)
354	(100)	96.0	45±3	3170	195, 681, 56
356	(100)	106.0	15±3	1400	522, 78, 46
540	(100)	96.0	25±3	750	240, 66, 9
164	(111)	38.6	20±3	3900	605, 656, 81
384	(111)	120.0	40±3	2000	696, 132, 57

**Table 2:** Sample name, surface orientation, laser treatment, mass and cut area of the CVD diamond samples.

Name	Orientation	Laser Treatment	Mass	Cut Area
			(mg)	(mm^2^)
1a	(100)	cut	32.5	14.4±0.4
1b	(100)	polish	33.6	14.4±0.4
2a	(110)	cut	35.6	5.7±0.2
2b	(110)	polish	33.3	5.7±0.2
3a	(111)	cut	39.5	7±0.2
3b	(111)	polish	65.7	6.7±0.2

**Table 3:** Main magnetic impurities content (in μg/g) measured by particle-induced X-ray emission (PIXE) of the natural diamond samples. The selected areas for the measurements are included. The spot area was 9μm${}^{2}$. MDL, minimum detectable limit.

Sample	Analyzed Area		Concentration			MDL	
		Fe	Co	Ni	Fe	Co	Ni
354	(0.5mm)${}^{2}$	0.17	0.046	0.16	0.03	0.03	0.03
	(160μm)${}^{2}$	0.08	<0.07	<0.07	0.05	0.04	0.05
356	spot	1.99	-	0.12	0.07	0.04	0.05
540	spot	0.57	-	0.11	0.06	0.04	0.04
164	(0.5mm)${}^{2}$	2.6	0.038	0.098	0.04	0.03	0.03
	(75μm)${}^{2}$	0.32	-	<0.17	0.2	0.2	0.2
384	(1 mm)${}^{2}$	0.84	0.28	0.35	0.08	0.08	0.07
	(400μm)${}^{2}$	0.8	<0.18	0.17	0.2	0.2	0.2

## Abbreviations

The following abbreviations are used in this manuscript:

CVD	Chemical vapor deposition
SQUID	Superconducting quantum interference device
PIXE	Particle-induced X-ray emission
